# Self-reported racial/ethnic discrimination and bronchodilator response in African American youth with asthma

**DOI:** 10.1371/journal.pone.0179091

**Published:** 2017-06-13

**Authors:** Sonia Carlson, Luisa N. Borrell, Celeste Eng, Myngoc Nguyen, Shannon Thyne, Michael A. LeNoir, Nadine Burke-Harris, Esteban G. Burchard, Neeta Thakur

**Affiliations:** 1 School of Medicine, University of California, San Francisco, San Francisco, California, United States of America; 2 Department of Epidemiology & Biostatistics, Graduate School of Public Health and Health Policy, City University of New York, New York, New York, United States of America; 3 Department of Medicine, University of California, San Francisco, San Francisco, California, United States of America; 4 Department of Allergy and Immunology, Kaiser Permanente-Oakland Medical Center, Oakland, California, United States of America; 5 Department of Pediatrics, University of California, Los Angeles, Los Angeles, California, United States of America; 6 Bay Area Pediatrics, Oakland, California, United States of America; 7 The Center for Youth Wellness, San Francisco, California, United States of America; 8 Department of Bioengineering and Therapeutic Sciences, University of California, San Francisco, San Francisco, California, United States of America; National and Kapodistrian University of Athens, GREECE

## Abstract

**Importance:**

Asthma is a multifactorial disease composed of endotypes with varying risk profiles and outcomes. African Americans experience a high burden of asthma and of psychosocial stress, including racial discrimination. It is unknown which endotypes of asthma are vulnerable to racial/ethnic discrimination.

**Objective:**

We examined the association between self-reported racial/ethnic discrimination and bronchodilator response (BDR) among African American youth with asthma ages 8 to 21 years (n = 576) and whether this association varies with tumor necrosis factor alpha (TNF-α) level.

**Materials and methods:**

Self-reported racial/ethnic discrimination was assessed by a modified Experiences of Discrimination questionnaire as none or any. Using spirometry, BDR was specified as the mean percentage change in forced expiratory volume in one second before and after albuterol administration. TNF-α was specified as high/low levels based on our study population mean. Linear regression was used to examine the association between self-reported racial/ethnic discrimination and BDR adjusted for selected characteristics. An interaction term between TNF-α levels and self-reported racial/ethnic discrimination was tested in the final model.

**Results:**

Almost half of participants (48.8%) reported racial/ethnic discrimination. The mean percent BDR was higher among participants reporting racial/ethnic discrimination than among those who did not (10.8 versus 8.9, p = 0.006). After adjustment, participants reporting racial/ethnic discrimination had a 1.7 (95% CI: 0.36–3.03) higher BDR mean than those not reporting racial/ethnic discrimination. However, we found heterogeneity of this association according to TNF-α levels (p-interaction = 0.040): Among individuals with TNF-α high level only, we observed a 2.78 higher BDR mean among those reporting racial/ethnic discrimination compared with those not reporting racial/ethnic discrimination (95%CI: 0.79–4.77).

**Conclusions:**

We found BDR to be increased in participants reporting racial/ethnic discrimination and this association was limited to African American youth with TNF-α high asthma, an endotype thought to be resistant to traditional asthma medications. These results support screening for racial/ethnic discrimination in those with asthma as it may reclassify disease pathogenesis.

## Introduction

Despite asthma prevalence variation according to sex over the life course, African Americans have one of the highest asthma prevalence and mortality rates in the U.S.[1] Overall, African American children experience higher prevalence of asthma (11.2%) than non-Hispanic whites (7.7%). This is also true for asthma mortality (0.23 per 1000 individuals in African Americans versus 0.13 per 1000 individuals in non-Hispanic whites) [1]. While there are well known risk factors for these disparities, psychosocial stress [2], including experiences of racial/ethnic discrimination [3], seems to be surfacing as an important risk factor. Experiences of racial discrimination are biased treatment associated with individual characteristics such as skin color [4]. A high proportion of minority youth (up to 88%) have reported experiencing racial discrimination [5].

However, the response to psychosocial stress is inconsistent [6,7] and, similarly, may also vary with experiences of racial/ethnic discrimination as the result of the heterogeneous nature of asthma. Asthma is no longer thought of as a single disease, but as a disorder composed of distinct types with varying pathophysiology. These varying types are endotypes of asthma and are thought to reflect a particular biologic mechanism linked to specific health outcomes such as inhaled corticosteroid response and frequent exacerbations [8]. Consequently, experiences of racial/ethnic discrimination may affect asthma outcomes differently according to these asthma endotypes.

A commonly used outcome of asthma is bronchodilator response (BDR), which aids in diagnosis [9], to assess responsiveness to inhaled corticosteroids, and as a predictor of future lung function [10]. Previous research has shown that a BDR ≥ 10% is associated with poor asthma control. Therefore, BDR is thought to be useful as a clinical tool to identify individuals at risk of poor asthma outcomes. Youth who experience racial/ethnic discrimination tend to have poor asthma control [3,11,12]. Thus, it is important to measure and assess the effects of discriminatory experiences related to race and/or ethnicity on BDR.

For this study, we focused on a moderate-to-severe asthma endotype that is neutrophilic and is associated with up-regulation of Tumor Necrosis Factor Alpha (TNF-α) [13]. This asthma endotype is characterized as having lower lung function [14]. It has been previously showed that even within a moderate-to-severe asthma group, a subgroup characterized by elevated TNF-α had higher reports of symptoms and excessive health care use compared to those with lower TNF-α [15]. Even within one endotype of asthma there are overlapping mechanisms [16], and thus, individuals may respond differently to the same trigger, including racial/ethnic discriminatory experiences.

**Objective**: We aimed to examine the association of self-reported racial/ethnic discrimination with BDR to albuterol among youth and whether this association varies with TNF-α levels.

## Materials and methods

### Study population

Participants for this study were enrolled through the Study of African Americans, Asthma, Genes & Environments (SAGE II) between 2008 and 2014. This parent study is a case-control study designed to examine the complex genetic and socio-environmental contributors to asthma prevalence, control and severity among minority children and adolescents. The SAGE II study recruited African American youth with and without asthma aged 8–21 years of age from urban regions in the San Francisco Bay Area. Asthma was defined as physician diagnosis and report of symptoms and medication use within the two years prior to recruitment [9]. To be eligible for the study, the survey respondent (participants <16 years old) or participant (≥16 years old) must have self-identified the parents and all four grandparents of the participant as African American. Those in the third trimester of pregnancy, a ≥10 pack-year smoking history, and current smokers were not eligible (S1 Table). All parents/participants provided appropriate written consent/assent. The University of California, San Francisco and each study site institutional review board (IRB) approved the SAGE II protocol (UCSF-IRB# 10–02877, Reference#155745).

### Assessment of self-reported racial/ethnic discrimination

Trained interviewers administered comprehensive questionnaires to the parents/caretakers of the participants to collect socio-demographic information, medical histories, and environmental exposure-related information. Interviewers were recruited from the same communities of potential participants and trained on respectively ascertaining sensitive information through questionnaires, spirometry, and biospecimen collection. The primary exposure for this analysis was self-reported racial/ethnic discrimination, ascertained using the Experiences of Discrimination (EOD) Questionnaire [17]. Consistent with a previous study [3], we included questions pertaining to our population: Have you ever experienced discrimination, been prevented from doing something, or been hassled or made to feel inferior, in any of the following situations because of your race, ethnicity, color, or language? (1) At School; (2) Getting medical care; (3) Getting services in a store or restaurant; and (4) On the street or in a public setting; with choice for each question of *Yes* or *No*. Experiences of discrimination were specified as none or any (affirmative answer to at least one situation).

### Assessment of biomarkers

Biomarkers were measured in stored frozen (-80°C) plasma specimens with storage times ranging from 3.1–9.5 years. Specimens were stored in multiple aliquots to minimize freeze-thaw cycle. TNF-α has been shown to remain stable over prolonged storage periods [18]. TNF-α was measured using a Magnetic Luminex Performance Assay from R&D systems in duplicate (n = 29) or triplicate form (n = 4). We excluded 16 individuals with failed assay (n = 5) or >10% variation in duplicate/triplicate value (n = 11). For individuals with x-10% variation in measured values (n = 8), we randomly selected one duplicate value to include as the measured value. Averages of remaining duplicate/triplicate values were used to determine the final measured TNF-α level for each individual. Storage time of TNF-α was added as a covariate and calculated based on date of recruitment and biomarker processing time. Consistent with previous studies [19,20], we classified individuals as TNF-α high and low based on being above or below our study population mean of 1.42 pg/ml.

### Covariates

Informed by previous studies, age [1], sex [21], *in utero* smoke exposure [22] (i.e., maternal smoking during pregnancy), socioeconomic status [23], body mass index (BMI) [24], early life exposure to daycare [25], and African ancestry [26] were considered as potential confounders. We used maternal educational attainment as a stable measure of socioeconomic status [3] and categorized as less than high school graduate, high school graduate, and some college or greater. Body mass index (BMI) was specified as BMI percentiles obtained using sex- and age- specific growth curves [27]. Estimates of African ancestry were obtained for each participant using an unsupervised analysis in ADMIXTURE assuming three ancestral populations. We used reference haplotypes from European and African individuals from HapMap phase II [28].

For this analysis, we included a measurement of baseline lung function and the report of asthma controller medications. Baseline lung function was measured using spirometry per American Thoracic Society guidelines. We used an individual’s percent of predicted force expiratory volume per one second (FEV_1_) measurement with a cutoff of 80%. The brief medication questionnaire [29] was used to ascertain reported controller medications use to ascertain asthma control. The National Heart, Lung, and Blood Institute’s (NHLBI) definition of asthma control is a composite score and the accepted standard to measure control [9]. Asthma control was derived from information collected through a modified version of the 1978 American Thoracic Society–Division of Lung Diseases Epidemiology Questionnaire [30] on symptoms, nighttime awakening, interferences with normal activities, and rescue medication use during the week prior to participant recruitment and interview and lung function measurements. Asthma control was defined for our analysis purposes as Controlled, Partially Controlled, or Poorly Controlled [3,22,25]. Controller medication use was defined as the report of inhaled corticosteroid, leukotriene inhibitor, or long-acting-beta agonist in the two weeks prior to recruitment. Finally, recruitment site was also considered as a covariate.

### Pulmonary function measures and bronchodilator response

The primary outcome for this study was maximal BDR to albuterol. All asthma medications were held for 12 hours before spirometry. Per the American Thoracic Society recommendations, pulmonary function was measured before albuterol administration and then repeated 15 minutes after administration of four puffs of albuterol (90 μg per puff) [31]. Spirometry was repeated a third time after a second dosage of albuterol (two puffs if < 16 years old or four puffs if ≥ 16 years old) [32]. We assessed the maximal BDR as the mean percentage change in measured FEV_1_ before and after albuterol administration, using the post-albuterol spirometry with the maximal change. For analytical purposes, BDR was specified as a continuous variable.

By 2014, there were 1009 eligible participants with asthma and stored biospecimens in SAGE II. Participants were excluded from the analysis if they were missing self-reported racial/ethnic discrimination questions (n = 194), variables related to SES (n = 20), environmental exposure data (daycare attendance and *in utero* smoke exposure; n = 45), pulmonary function measures (n = 69), or had inconclusive or missing TNF-α measurements (n = 83), or other covariate information (n = 22). These exclusions yielded an analytical sample size of 576. When comparing records for excluded and included participants, excluded participants were older (14.5 versus 13.5 years, p = 0.008), more likely to report *in utero* tobacco smoke exposure (24.1 versus 18.6%, p = 0.034), and less likely to have mothers with higher education (50.7 versus 60.9%, p < 0.001).

### Statistical analysis

Descriptive statistics for cases according to reports of self-reported racial/ethnic discrimination were calculated. Significance differences and associations were determined using Student t-test and Kruskal-Wallis test according to whether continuous variables were normally distributed or not, respectively, and chi-square tests for categorical variables. Covariates associated with BDR (p < 0.2) were included in the final model. We used linear regression to estimate the association between self-reported racial/ethnic discrimination and BDR before and after controlling for selected covariates. To determine whether this association varies with TNF-α level, an interaction term between self-reported racial/ethnic discrimination and TNF-α was tested in the final model. Significance for main effects was determined at 0.05 and for interaction terms at 0.10. All analyses were conducted with R 3.1.2 [33].

## Results

### Baseline study characteristics

Selected characteristics of participants according to self-reported racial/ethnic discrimination are displayed in Table 1. Almost half (48.8%) of our participants reported experiences of racial/ethnic discrimination in any setting at some point in their life. When compared with youth who do not report experiencing racial/ethnic discrimination, participants with self-reported experiences of racial/ethnic discrimination were older (median age 15.4 versus 12.1 years, p < 0.001), more likely to be exposed to *in utero* smoke (22.1 versus 15.3%, p-value = 0.036) and had mothers with higher levels of educational attainment compared with those who did not reported racial/ethnic discrimination (67.3 versus 54.9%, p-value = 0.008). Participants who reported racial/ethnic discrimination were more likely to have very poorly controlled asthma (50.2 versus 33.9%; p < 0.001). Moreover, the mean percent BDR was higher among those reporting racial/ethnic discrimination (10.8, SD 9.8) than among those not reporting racial/ethnic discrimination (8.9, SD 7.8; p = 0.006). There was no association between TNF-α and self-reported racial/ethnic discrimination.

**Table 1:** Selected characteristics of participants with asthma according to self-reported racial/ethnic discrimination in SAGE II (2006–2014).

Characteristic	Racial/ethnic Discrimination^a^		
	None	Any	
	No. (%)^b^	No. (%)^b^	p -value
**Prevalence**	295 (51.2)	281 (48.8)	
**Age**, median (IQR)	12.1 (4.8)	15.4 (5.5)	**< 0.001**
**Sex**, male	160 (54.2)	151 (53.7)	0.904
**Tobacco Exposure**			
Current	82 (28.4)	88 (31.5)	0.410
*In-Utero*	45 (15.3)	62 (22.1)	**0.036**
**Daycare Attendance**			
Yes	204 (69.2)	208 (74.0)	0.196
No	91 (30.8)	73 (26.0)	
**Education Level**^c^			
Some HS	35 (11.9)	32 (11.4)	**0.008**
HS Graduate	98 (33.2)	60 (21.4)	
Some College	162 (54.9)	189 (67.3)	
**%African Ancestry**, mean (SD)	77.3 (12.7)	78.9 (11.0)	0.298
**Atopy**			
None	104 (35.9)	106 (38.0)	0.976
Rhinitis or Eczema	119 (41.0)	102 (36.6)	
Both	67 (23.1)	71 (25.4)	
**Asthma Control**			
Controlled	110 (37.3)	59 (21.0)	**< 0.001**
Not well Controlled	85 (28.8)	81 (28.8)	
Very Poorly Controlled	100 (33.9)	141 (50.2)	
**Controller medication use**			
No	178 (60.3)	190 (67.6)	0.069
Yes	117 (40.0)	91 (32.4)	
**TNF-**α level			
High	142 (48.1)	136 (48.4)	0.950
Low	153 (51.9)	145 (51.6)	
**% Bronchodilator Response,** mean (SD)	8.9 (7.8)	10.8 (9.4)	**0.006**

*Definition of Abbreviations*: HS = high school, SAGEII = Study of African Americans, Asthma, Genes and Environment, SES = socioeconomic status

^a^ Racial/ethnic discrimination was categorized as None (negative answer to all 4 situations) or Any (affirmative answer to one or more situations)

^b^Values are reported as numbers (percentages) unless otherwise specified

^c^Refers to the education level of the participant’s mother

### Bronchodilator response and self-reported racial/ethnic discrimination

Participants who reported any racial/ethnic discrimination had a 1.7 (95%CI 0.36–3.03) greater mean percent BDR compared with children not reporting racial/ethnic discrimination after adjusting for sex, age, maternal education, recruitment center, *in utero* smoke exposure, daycare attendance, baseline lung function, controller medication use, African ancestry, TNF-α mean, and biomarker storage time (Table 2). However, a significant heterogeneity of this association was observed according to TNF-α status (High/Low; p-interaction = 0.040). For participants in the TNF-α high group, those reporting racial/ethnic discrimination had a 2.78 (95%CI: 0.79–4.77) greater mean percent BDR to albuterol than those not reporting racial/ethnic discrimination. This association was not observed among those in the TNF-α low group (S1 Fig). Selected characteristics of participants with TNF-α high and low asthma are reported in the supplement and displayed in S2 Table.

**Table 2:** Mean difference in bronchodilator response^a^ and 95% CI for reports of racial/ethnic discrimination and according to TNF-α status for SAGE II participants with asthma (2006–2014).

		TNF-α Status^c^	
	Adjusted^b^	Low^b^	High^b^
**Racial/ethnic Discrimination**			
**Never**	0	0	0
**Any**	1.70 (0.36, 3.03)	0.78 (-1.07, 2.63)	2.78 (0.79, 4.77)

^a^ Bronchodilator response: mean percentage change in measured FEV_1_ before and after albuterol administration, using the post-albuterol spirometry with the maximal change.

^b^Adjusted for sex, age, maternal education, recruitment center, *in utero* smoke exposure, daycare attendance, baseline lung function, controller medication use, African ancestry, TNF-α mean, and biomarker storage time.

^c^P-interaction = 0.04

## Discussion

In this study, we observed an association between self-reported racial/ethnic discrimination and mean BDR. However, the significant increased mean BDR with those self-reporting racial/ethnic discrimination was observed only among participants in the TNF-α high group. Our results corroborated previous studies suggesting that self-reported racial/ethnic discrimination as a psychosocial stressor may affect health in youth, including asthma outcomes [2,3,12]. In contrast to a previous study which showed that psychosocial stress reduces BDR [34], we observed an increase in BDR in participants with reports of self-reported racial/ethnic discrimination and TNF-α high level. This supports the theory that asthma is heterogeneous and that this heterogeneity extends to the endotypes already identified, such as TNF-α asthma [16].

Our study shows that screening for experiences of racial/ethnic discrimination, as a type of psychosocial stress, may be important among those with moderate-severe asthma. This is clinically relevant as different treatments or interventions may be applied to this difficult to control group. There is evidence that adjunct socio-behavioral interventions to traditional asthma management improve outcomes [35]; however, these interventions are perceived as time and labor intensive. By identifying a risk factor profile that includes measures of racial/ethnic discriminatory experiences and inflammatory biomarkers, we may be better able to screen and identify individuals who are most susceptible to this type of psychosocial stress, and thus, more likely to benefit from such therapy. In addition, identification of such profile provides illumination on the various biological mechanisms to the development of TNF-α high asthma.

The different responses to medication among individuals thought to represent one endotype of asthma generate speculation on mechanisms for the various asthma endotypes [8,36,37]. One pathway may involve inflammatory and neuro-endocrine mechanisms that lead to different asthma endotypes [38]. These pathways may explain the variation in response to stress brought on by childhood upbringing, environment, genetics and race/ethnicity. Biomarkers of stress involved in systemic inflammation, such as TNF-α, have been shown to be elevated in acute asthma exacerbations in comparison to individuals with well controlled asthma [39]. Additionally, individuals reporting racial/ethnic discrimination have been shown to have elevated levels of cytokines, including TNF-α, compared with those not reporting racial/ethnic discrimination [40]. In asthma, psychosocial stress secondary to experiences of racial/ethnic discrimination may enhance airway inflammation by modulating immune cell function through hormonal pathways [38,41]. One mouse model shows how social stress potentially alters lung function: stress led to increased levels of TNF-α and decreased drug response to inhaled corticosteroids [41]. A similar pathway has been described for TNF-α-high-asthma, which has been known to be severe and non-responsive to asthma medications [15].

Unmeasured factors related to discriminatory experiences may account for some of the association seen with bronchodilator response. Individuals experiencing racial/ethnic discrimination are likely to be from communities that are marginalized and the most affected by structural racism. This includes living in areas exposed to higher levels of indoor and outdoor air pollution, substandard housing, and exposure to community violence; all factors that are also associated with segregated neighborhoods and associated socioeconomic disadvantages. In fact, racial segregation has been independently shown to be a fundamental cause of health disparities in health [42], including asthma.[43]

There are several other limitations in the study. First, the cross-sectional design of our study limits our ability to identify causal relationships or reverse causation between our exposure and outcome. We are unable to determine if the observed relationship between asthma and reports of racial/ethnic discrimination is actually the result of asthma itself as a socially stigmatized status [44,45]. Second, we observed that the response to albuterol varied greatly in our study population and ranged from -10% (a negative response) to 105% increase from the pre-albuterol FEV_1_. This variability in bronchodilator response is common [46] and similar to what has been observed in other study populations [34]. Our findings of increased bronchodilator response in those with high TNF-α high asthma plus self-reported racial/ethnic discrimination should be taken in this context. While the exclusion of outliers (those with bronchodilator responses greater than 60%) reduces the standard deviations, similar results to the ones we presented here were observed, suggesting the association between BDR and reports of racial/ethnic discrimination were not influenced or driven by the outliers. Third, because asthma is an inflammatory disease, TNF-α, a marker of inflammation may be elevated in youth with asthma as a result of the underlying disease and altered by controller medications, which have anti-inflammatory properties. Our analyses included controller medication use and a marker of lung function severity as covariates to help address these issues. Fourth, we excluded 433 participants due to missing data mostly for discrimination measures, pulmonary function measures, or TNF-α measurements. However, participants were selected based on disease status and not on reports of racial/ethnic discrimination and/or spirometry measures, and thus, it is unlikely that these exclusions have biased our results. Finally, the discrimination questionnaire tool we used has been validated in adults, but not in children. Despite this limitation, the questions we included overlap with those previously used in instruments validated for children [5,47]. In addition, we previously found that racial/ethnic discrimination, ascertained using these questions, was associated with asthma related outcomes in a pediatric population [3]. Despite these limitations, these data were obtained from a well conducted case control study [3] collecting a wide breadth of sociodemographic, medical history and environmental exposure data.

Future studies should aim for the development of an advanced tool to assess experiences of racial/ethnic discrimination and other psychosocial stressors in youth. Furthermore, studies addressing discrimination across the lifespan could give better insight to how asthma outcomes change based on acute versus chronic psychosocial stressor exposure and allow interventions to take place with follow up to examine changes in asthma outcomes. Finally, other asthma endotypes such as atopic asthma and obese asthma would be worth examining to observe their response to racial/ethnic discrimination as a form of psychosocial stress. Strengthening risk profiling abilities will allow health care providers to identify those at highest risk and intervene earlier in children’s lives, when they are most susceptible to social stress.

## Conclusions

Our study confirms previous findings that psychosocial stress impacts asthma outcomes in children [2,3]. We found BDR to be increased in participants who self-reported racial/ethnic discrimination with this increase being greater among African American youth with TNF-α high asthma, an asthma type thought to be resistant to traditional asthma medications. This finding is clinically important, as those who are at risk of poor asthma outcomes and were previously thought to be unresponsive to asthma medications [15] may actually be responsive and benefit from adjunct behavioral and/or environmental interventions. These results support the need to screen for racial/ethnic discriminatory experiences among those with moderate-severe asthma as it may help to reclassify asthma type and identify more precise treatments for high-risk population.

## Supporting information

S1 Table Eligibility criteria for participation for SAGE II asthma cases. (DOCX)

S2 Table Selected characteristics^a^ of participants according to TNF-α status in SAGE II (2006–2014). *Definition of Abbreviations*: HS = high school, SAGEII = Study of African Americans, Asthma, Genes and Environment. ^a^Values are reported as numbers (percentages) unless otherwise specified. ^b^Refers to the education level of the participant’s mother. ^c^Racial/ethnic discrimination score was categorized as None (negative answer to all 4 situations) or Any (affirmative answer to one or more situations). ^d^Report of any asthma controller medication use in the 2 weeks prior to recruitment including inhaled corticosteroids, long acting beta agonist, and/or montelukast.
(DOCX)

S1 Fig Bronchodilator response (%) by level of reported racial/ethnic discrimination (None/Any) stratified by TNF-α status for participants with asthma from SAGE II recruited from 2006–2014. Means are adjusted for sex, age, maternal education, recruitment center, *in utero* smoke exposure, daycare attendance, baseline lung function, controller medication use, African ancestry, and biomarker storage time.
(TIF)
