# Supplementary material for: Self-reported racial/ethnic discrimination and bronchodilator response in African American youth with asthma
Source: PLoS One. 2017 Jun 13;12(6):e0179091. doi: 10.1371/journal.pone.0179091 (PMC5469454; doi:10.1371/journal.pone.0179091)
Supplement: S1 Table — (DOCX) [file pone.0179091.s001.docx]

| **S1 Table. Eligibility Criteria for Participation for SAGE II Asthma Cases.** | |
| --- | --- |
| **Criterion** | **Asthma Cases** |
| Age between 8 and 21 years old | Yes |
| All four grandparents self-identified as African American (SAGE II) | Yes |
| History of physician-diagnosed asthma | Yes |
| Symptoms of wheezing or shortness of breath | Yes |
| No respiratory infections for ≥ 6 weeks (clinical stability) | Yes |
| No asthma exacerbations for ≥ 6 weeks (clinical stability) | Yes |
| Less than 10 pack year smoking history and no smoking in the last year | Yes |
| If pregnant, < 3rd trimester | Yes |
| No history of other lung diseases or other chronic illnesses | Yes |
