# Supplementary material for: Self-reported racial/ethnic discrimination and bronchodilator response in African American youth with asthma
Source: PLoS One. 2017 Jun 13;12(6):e0179091. doi: 10.1371/journal.pone.0179091 (PMC5469454; doi:10.1371/journal.pone.0179091)
Supplement: S2 Table — Definition of Abbreviations: HS = high school, SAGEII = Study of African Americans, Asthma, Genes and Environment. aValues are reported as numbers (percentages) unless otherwise specified. bRefers to the education level of the participant’s mother. cRacial/ethnic discrimination score was categorized as None (negative answer to all 4 situations) or Any (affirmative answer to one or more situations). dReport of any asthma controller medication use in the 2 weeks prior to recruitment including inhaled corticosteroids, long acting beta agonist, and/or montelukast. (DOCX) [file pone.0179091.s002.docx]

| **S2 Table. Selected Characteristics**^a^ **of Participants according to TNF-α status in SAGE II (2006-2014).** | | | |
| --- | --- | --- | --- |
|  | **Low TNF-α** | **High TNF-α** | **p-value** |
| **Prevalence** | 298 (51.7%) | 278 (48.3) |  |
| **Age**, median (IQR) | 14.1 (6.2) | 12.8 (5.4) | **0.012** |
| **Sex**, male | 138 (46.3) | 173 (62.2) | **0.001** |
| **Tobacco Exposure**  Current  *In-Utero* | 82 (27.7)  49 (16.4) | 88 (32.4)  58 (20.9) | 0.227  0.173 |
| **Daycare Attendance**  Yes  No | 215 (72.1)  83 (27.9) | 197 (70.9)  81 (29.1) | 0.733 |
| **Education Level**^b^  Some HS  HS Graduate  Some College | 30 (10.1)  77 (25.8)  191 (64.1) | 37 (13.3)  81 (29.1)  160 (57.6) | 0.092 |
| **Discrimination**^c^  Never  Any | 153 (51.3)  145 (48.7) | 142 (51.1)  136 (48.9) | 0.950 |
| **%African Ancestry**, mean (SD) | 77.6 (12.6) | 78.6 (11.2) | 0.930 |
| **Atopy**  None  Rhinitis or Eczema  Both | 97 (33.2)  120 (41.1)  75 (25.7) | 113 (40.8)  101 (36.5)  63 (22.7) | 0.094 |
| **Controller medication use**^d^  No  Yes | 190 (63.8)  108 (36.2) | 178 (64.0)  100 (36.0) | 0.946 |
| **Asthma Control**  Controlled  Not well Controlled  Very Poorly Controlled | 96 (32.2)  92 (30.9)  110 (36.9) | 73 (26.3)  74 (26.6)  131 (47.1) | **0.018** |
| **% Bronchodilator Response,** mean (SD) | 9.3 (8.1) | 10.4 (9.1) | **0.013** |
| *Definition of Abbreviations:* HS = high school, SAGEII = Study of African Americans, Asthma, Genes and Environment  ^a^Values are reported as numbers (percentages) unless otherwise specified  ^b^Refers to the education level of the participant’s mother  ^c^Discrimination score was categorized as Never (negative answer to all 4 situations); Any (affirmative answer to one or more situations)  ^d^Report of any asthma controller medication use in the 2 weeks prior to recruitment including inhaled corticosteroids, long acting beta agonist, and/or montelukast | | | |
