# Supplementary figures and images for: Self-reported racial/ethnic discrimination and bronchodilator response in African American youth with asthma
Source: PLoS One. 2017 Jun 13;12(6):e0179091. doi: 10.1371/journal.pone.0179091 (PMC5469454; doi:10.1371/journal.pone.0179091)

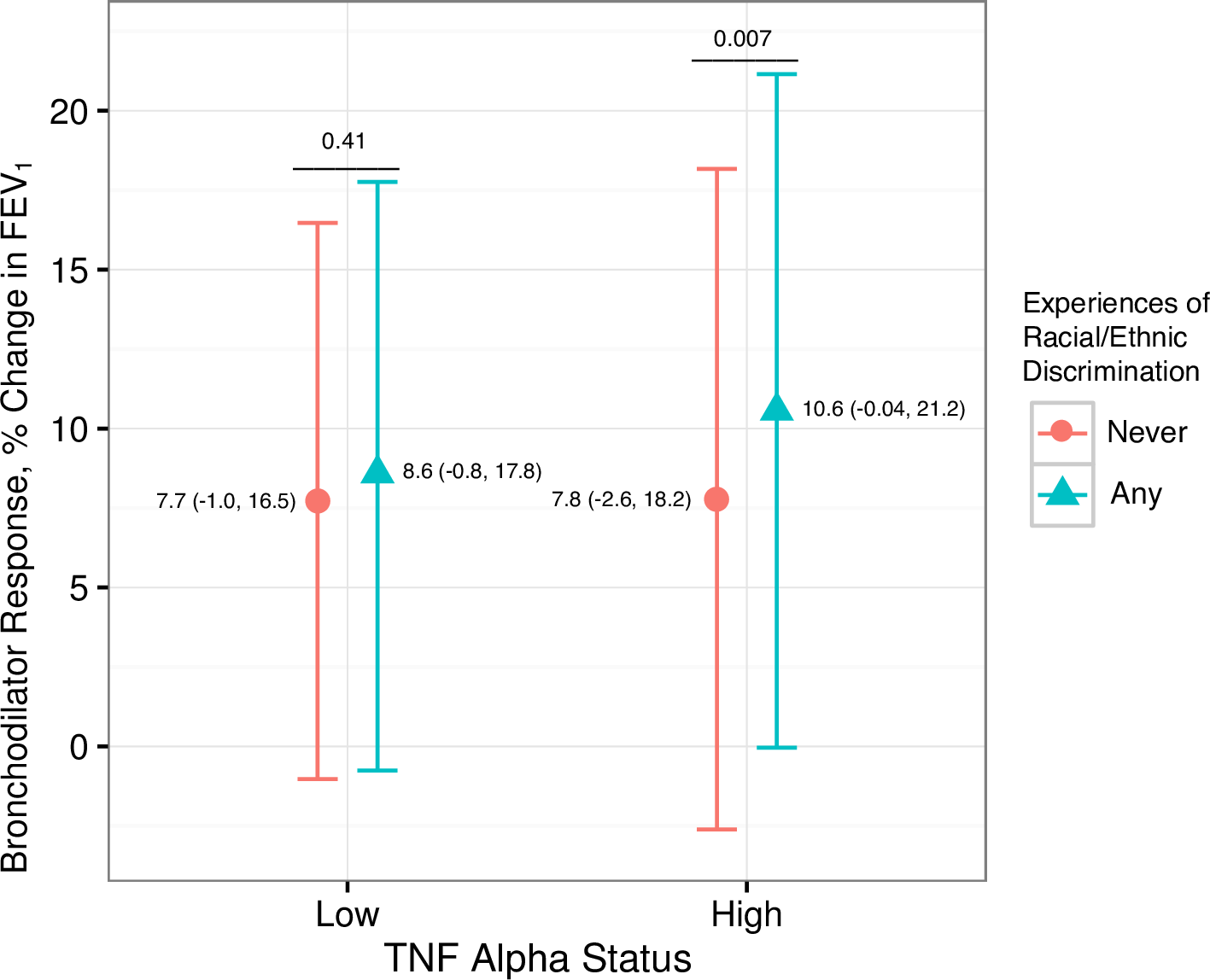

Supplement: S1 Fig — Means are adjusted for sex, age, maternal education, recruitment center, in utero smoke exposure, daycare attendance, baseline lung function, controller medication use, African ancestry, and biomarker storage time. (TIF) [file pone.0179091.s003.tif]
